# Safety and Efficacy of Direct Antiviral Agents for Hepatitis C in Patients with Malignancies Other Than Liver Cancer: A Case Series

**DOI:** 10.3390/pathogens11080860

**Published:** 2022-07-29

**Authors:** Fabian Patauner, Maria Stanzione, Gianfranca Stornaiuolo, Veronica Martone, Roberta Palladino, Nicola Coppola, Emanuele Durante-Mangoni, Rosa Zampino

**Affiliations:** 1 Department of Advanced Medical and Surgical Sciences, University of Campania Luigi Vanvitelli, 80138 Napoli, Italy; fabian@patauner.it (F.P.); veronicamartone@gmail.com (V.M.); rosa.zampino@unicampania.it (R.Z.); 2 Department of Mental Health and Public Medicine, Infectious Diseases Unit, University of Campania Luigi Vanvitelli, 80138 Napoli, Italy; maria.stanzione@unicampania.it (M.S.); gianfranca.stornaiuolo@unicampania.it (G.S.); roberta.palladino@studenti.unicampania.it (R.P.); nicola.coppola@unicampania.it (N.C.); 3 Department of Precision Medicine, University of Campania Luigi Vanvitelli, 80138 Napoli, Italy; 4 Unit of Infectious and Transplant Medicine, AORN Ospedali Dei Colli-Monaldi Hospital, 80131 Naples, Italy

**Keywords:** HCV, DAA, cancer, chemotherapy, radiotherapy, cirrhosis

## Abstract

(1) Background: direct-acting antivirals (DAA) are the current standard of care for chronic hepatitis C. Oncologic patients remain among the most difficult-to-treat subgroups of hepatitis C virus (HCV)-infected patients due to their clinical frailty and complex therapeutic protocols received. (2) Methods: we retrospectively collected and analysed clinical data of 30 consecutive patients treated with DAA, between 2015 and 2022, for chronic HCV infection in the context of oncologic disease. (3) Results: most patients were females (63.3%), median age was 67 years, HCV genotype 1 was prevalent (60%), and median HCV RNA levels were 2.2 × 10^6^ IU/mL. The most common malignancy was breast cancer (37%), and the chief oncologic drugs co-administered with DAAs were tamoxifen, platinum derivatives, cyclophosphamide, paclitaxel, rituximab and doxorubicin. Overall, 50% of patients had chronic hepatitis. A total of 76.7% underwent a sofosbuvir-based treatment. Sustained virological response 12 weeks after the end of therapy (SVR12) was reached in all patients. After SVR12, two patients died. DAA treatment was well tolerated; no patients had to stop DAA treatment or showed any adverse event or drug-drug interaction specifically attributable to DAAs. (4) Conclusions: DAA treatment should be promptly offered to oncologic patients with chronic hepatitis C in order to achieve aminotransferase normalization and viremia control, making antineoplastic therapy feasible and safe.

## 1. Introduction

Hepatitis C virus (HCV) infection is still prevalent in the general population and is often detected by chance in patients unaware of their infection [1,2]. This often occurs in the context of the diagnostic workup for other diseases, including malignancies [3,4].

Direct-acting antiviral (DAA) agents are the current standard of care for chronic hepatitis C and are safe and effective in more than 95% of treated patients [1,5,6,7]. Drug–drug interactions represent a common issue requiring particular attention during treatment [8,9,10]. DAAs have been largely employed in the oncology setting and were shown to be safe and effective mostly in patients with hepatocellular carcinoma and lymphomas [10,11,12,13,14]. Indeed, chronic hepatitis C can complicate cancer treatment in patients with solid or hematological malignancies that are not related to HCV infection. High viral load, often a sign of immunosuppression, and/or high alanine aminotransferase (ALT) levels, possibly due to liver toxicity, can preclude the completion of full antineoplastic protocols. Specifically, HCV infection often causes serious liver disease with advanced inflammation and/or severe fibrosis [15,16,17,18].

Accordingly, oncologic patients remain among the most difficult-to-treat subgroups of HCV-infected patients due to their clinical frailty and the complex therapeutic protocols received. Further experience is sorely needed in this setting.

We retrospectively collected and analysed clinical data of patients treated for chronic HCV infection in the context of oncologic disease. Our aim was to assess the safety and efficacy of DAA therapy and patients’ tolerability in this special subgroup and in the presence or absence of antineoplastic therapy.

## 2. Patients and Methods

### 2.1. Patients

Data of consecutive 30 patients with chronic hepatitis C and a malignancy, followed at the Unit of Infectious amd Transplant Medicine or the Unit of Infectious Diseases, University of Campania “L. Vanvitelli”, between 2015 and January 2022, were included in this retrospective analysis. Patients made up a relatively heterogeneous group as they were referred by oncologists of different hospitals in our region and in different moments of their oncologic history and treatment.

Upon the first observation, patients underwent a complete physical exam, full liver function tests, blood cell count, assessment hepatitis B virus (HBV) and human immunodeficiency virus (HIV) markers, quantitative HCV-RNA, HCV genotype and liver ultrasound scan. For each patient, the body mass index (BMI: kg/m^2^) was calculated.

Liver fibrosis was evaluated by transient elastography (TE, FibroScan, EchoSens, Paris, France), while clinical cirrhosis was diagnosed in patients with a clear clinical presentation (i.e., splenomegaly, esophageal varices, ascites).

### 2.2. Antiviral Treatment 

Patients were treated according to the ongoing modifications of EASL guidelines and DAA availability in Italy [1,19,20,21]. 

A careful drug history was taken, and a consonant adjustment of treatments was carried out, when needed and possible, according to the European Association for the Study of the Liver (EASL) guidelines and HEP Drug Interactions, University of Liverpool website (http://hepdruginteractions.org).

Laboratory tests were performed by hospital standard procedures. Patients were followed up once a month for the duration of DAA treatment and at least 3 months after the end of treatment.

Response to antiviral treatment was defined as the sustained virological response (SVR = HCV RNA undetectable) 12 weeks (SVR12) after the end of therapy. Relapse was defined as the reappearance of serum HCV RNA after the end of DAA treatment.

Personal and clinical data were managed in agreement with the Declaration of Helsinki and the General Data Protection Regulation (679/2016) and were approved by our Ethical Committee of Università della Campania L. Vanvitelli (protocol code 21399/2021). Informed consent was obtained from all subjects involved in the study.

Data analyses were largely descriptive and included the presentation of categorical data as number and percent and numerical data as median and range.

## 3. Results

### 3.1. Baseline

The general clinical features of each patient included in the study are reported in Table 1. Most patients were females (63.3%), had a median age of 67 years (range 44–87) and a median BMI of 25.5 (range 21–34). Eighty percent of patients presented with solid cancer, while 20% were affected by a hematological malignancy. The most common malignancy was breast cancer (11 cases, 37%), and the chief oncologic drugs co-administered with DAA were tamoxifen, platinum derivatives, cyclophosphamide, paclitaxel, rituximab and doxorubicin (Table 1). 

The median estimated glomerular filtration rate (by Modification of Diet in Renal Disease, MDRD study equation) was 83 mL/min × 1.73 m^2^ (range 46–190). Many co-morbidities were also present, with a median Charlson Comorbidity Index (CCI) of 7 (range 2–11).

In terms of liver disease stage, 50% of patients had chronic hepatitis, and 50% had cirrhosis. Only one patient had an HBV co-infection already on specific antiviral treatment. HCV genotype 1 was prevalent (60%) and median HCV RNA levels were 2.2 × 10^6^ IU/mL (range 3.9 × 10^3^–96.1 × 10^6^).

At baseline, 53.3% of patients showed increased ALT levels (median 57 U/L, range 10–200 U/L). In addition, 90% of patients were naive to antiviral treatment, and 10% were previously non-responder to peg-interferon plus ribavirin.

Regarding DAA treatment, 76.7% underwent a sofosbuvir-based treatment, and 23.3% underwent a non-sofosbuvir-based treatment; in only 4 patients, ribavirin was added to the treatment regimen. During DAA treatment, 80% of patients were exposed to chemotherapy, and as many as 33.3% were exposed to radiotherapy.

### 3.2. Treatment Results

ALT levels became normal in 93.3% of patients (median 21.5 U/L range 10–93.1 U/L) after 1 month of DAA treatment and in 97% of patients after 2 months of DAA treatment. 

All patients obtained a documented SVR12. A total of 19 (63%) patients were followed up for a longer time and were assessed at 24 weeks after DAA treatment; of these, 1 patient relapsed, whilst 18 maintained the SVR. Two patients (7%) died, and seven patients (23%) were lost to follow-up after reaching SVR12; furthermore, two patients (7%) had not yet reached 24 weeks after the end of therapy. Patients died for reasons unrelated to DAA treatment but for a rapid worsening of oncologic disease. 

No HBV reactivation was observed in the HBV-HCV coinfected patient, who continued his HBV-directed antivirals.

DAA treatment was well tolerated; no patients had to stop DAA treatment or showed any adverse event specifically attributable to DAA. In particular, no specific adverse event could be identified when DAA was added to ongoing oncologic therapy. Only patients treated with ribavirin (*n* = 4) developed mild anemia, with a median hemoglobin 1 month after starting treatment of 12.2 g/dL. Furthermore, no drug-drug interactions were observed. There were no differences in safety and efficacy between the different antiviral regimens.

As regards the oncologic outcome, among patients with ALT above the UNL at baseline, 11 (68.8%) had stable disease, 3 (18.7%) had progressive disease, and 2 (12.5%) died. Among patients with normal ALT values at baseline, 12 (85.7%) had stable disease, and 2 (14.3%) had progressive disease.

## 4. Discussion

Chronic hepatitis C remains highly prevalent in specific geographic areas of the world, including the Campania region of southern Italy [1,22]. 

Patients with malignancy and HCV infection represent a special population with a greater risk of oncologic treatment delay, dose modification and longer treatment duration as previously reported in a specific cancer population [23]. At the moment, no specific treatment indications are provided in the current EASL guidelines [1]. In real life, hepatologists often face patients who become aware of HCV infection during the diagnostic workup for malignancy, but few data are available regarding the outcomes of antiviral treatment in this subgroup.

DAA treatment is generally safe, effective and well tolerated, and only a limited life expectancy for non-liver-related co-morbidities is now considered a contraindication to therapy [1]. In oncologic patients, this issue is very important, as short life expectancy is possible in advanced disease. Thus, a careful prognostic stratification of the malignancy is necessary to appropriately consider DAA treatment as already reported in patients with hematological cancers [24]. 

In many cases, HCV-related elevation of liver enzymes contraindicates or complicates cancer chemotherapy [25]; antiviral treatment, with consequent fast ALT normalization, allows for higher dose regimens and access to investigational cancer therapies, possibly increasing the chances of an oncologic cure [26,27].

DAA treatment was safe and effective in our 30 oncologic patients. They were referred to us during a period of 6 years and were followed up and treated with different protocols according to the ongoing modifications of EASL guidelines and DAA availability in Italy. However, all treatments and protocols were well tolerated and largely achieved a sustained virological response, allowing for oncologic treatment to be carried out without specific modifications. A careful evaluation of drug-drug interactions before starting antiviral therapy allowed us to proceed with oncologic treatment without adverse events or the need to stop treatment. Previous experience also showed good tolerability of DAA treatment during chemotherapy [10,26].

Half of our patients were treated with newer DAA schedules (sofosbuvir/velpatasvir and pribentasvir/glecaprevir) with a good antiviral response and no adverse drug reactions. These regimens are simpler, shorter and with fewer drug-drug interactions than previous ones, encouraging antiviral treatment start.

Despite the low number of patients and the not homogeneous follow-up, our findings suggest oncologists should not be concerned when treating patients with HCV infection. However, a longer follow-up could be useful in this category of patients to highlight possible late viral reactivations or clinical flares related to the immunosuppressive state.

In conclusion, in the absence of specific contraindications or limited life expectancy, DAA treatment should be promptly offered to oncologic patients with chronic hepatitis C in order to achieve ALT normalization and viremia control, making antineoplastic therapy feasible and safe.

## Figures and Tables

**Table 1:** General characteristics of the study group.

Patient N°	Age	Sex	CCI	Malignancy	Oncologic Treatment during DAA	Radiotherapy during DAA	HCV-RNA Baseline	HCV Genotype	ALT Baseline	Fibrosis *	Antiviral Treatment	Oncologic Outcome ^	Virologic Outcome
1	71	M	6	Prostate cancer	LEU	No	16700000	2	10	F1	SOF/VEL	SD	SVR 12
2	81	M	8	CLL	No	No	1885206	2	24	F3	SOF/VEL	SD	SVR 12
3	46	M	7	Lung cancer	No	Yes	142000	2	98	F3	SOF/VEL	PD	SVR 12
4	69	M	5	Lung cancer	CAR-PAC-PEM	No	800000	1	103	F3	SOF/VEL	SD	SVR 12
5	74	M	8	Prostate cancer	No	Yes	1200000	3	118	F4	GLE/PIB	SD	SVR 12
6	76	F	10	Colon cancer	OXA-CAP	No	4100000	2	200	F4	SOF/VEL	PD	SVR 12
7	78	F	11	Breast cancer	PAC, TRA, PER	Yes	45000000	1b	183	F4	SOF/VEL	PD	SVR 12
8	63	F	6	Breast cancer	CYC-EPI	Yes	800000	1a	71	F3	SOF/VEL	SD	SVR 12
9	59	M	8	Metastatic melanoma	NIV	No	3000000	1a	65	F2	GLE/PIB	SD	SVR 12
10	65	F	7	Breast cancer	EPI, CYC, PAC, TRA	No	67700000	2	104	Cl. Cirr	SOF/VEL	SD	SVR 12
11	77	F	10	Breast cancer	CYC	No	72000000	2	12	F0-F1	GLE/PIB	SD	SVR 12
12	65	F	6	Colon cancer	OXA- CAP	Yes	16000000	1	105	F3	3D	SD	SVR 12
13	63	M	5	Non-Hodgkin Lymphoma	R-CHOP	No	16400000	1	48	F0-F1	SOF/LDV	SD	SVR 12
14	64	F	8	Breast cancer	TAM	Yes	1200000	1	23	F0-F1	ELB/GR	SD	SVR 12
15	55	F	6	Non-Hodgkin Lymphoma	HSCT	No	9360000	1b	35	Cl. Cirr	SOF/VEL	SD	SVR 12
16	79	M	8	Multiple myeloma	BOR	No	461000	1b	42	F4	SOF/LDV	PD	SVR 12
17	87	M	9	Bladder cancer	No	Yes	302000	2a	144	Cl. Cirr	SOF/VEL	SD	SVR 12
18	57	F	4	Breast cancer	TAM	No	96100000	2a	40	F3	SOF+RBV	SD	SVR 12
19	63	F	5	Breast cancer	CYC	No	4500000	2a\2c	22	F2	SOF/VEL	SD	SVR 12
20	78	F	8	Breast cancer	TAM	No	169000	1b	47	Cl. Cirr	SOF/LDV	SD	SVR 12
21	77	F	8	Breast cancer	TAM	Yes	3900	1b	37	Cl. Cirr	3D + RBV	PD	SVR 12
22	69	F	7	Colon cancer	OXA-CAP	No	2450000	1b	98	Cl. Cirr	SOF/LDV	SD	SVR 12
23	68	F	4	Non-Hodgkin Lymphoma	R-CHOP	No	230000	4	21	F0-F1	SOF/LDV	SD	SVR 12
24	70	M	7	Prostate cancer	LEU	Yes	512000	1b	67	Cl. Cirr	3D + RBV	SD	SVR 12
25	66	F	4	Non-Hodgkin Lymphoma	R-CHOP	No	5200000	2a	32	F0-F1	SOF/VEL	SD	SVR 12
26	60	M	9	Lung cancer	CIS-ETO	No	4200000	1b	104	Cl. Cirr	SOF+SIM+RBV	PD	SVR 12
27	71	F	8	Breast cancer	TAM	No	2100000	1b	56	Cl. Cirr	SOF/LDV	SD	SVR 12
28	61	F	10	Uterine cancer	No	Yes	2300000	1b	65	Cl. Cirr	SOF/LDV	PD	SVR 12
29	60	F	7	Gastric cancer	IMA	No	1230000	1b	58	F4	SOF/LDV	SD	SVR 12
30	44	F	2	Breast cancer	TAM	No	1200000	1b	30	F1	SOF/VEL	SD	SVR 12

3D, ombitasvir + paritaprevir + ritonavir + dasabuvir; ALT, alanine aminotransferase; BOR, bortezomib; CAP, capecitabine; CAR, carboplatin; CCI, Charlson Comorbidity Index; CIS, cisplatin; Cl. Cirr, clinical cirrhosis; CLL, chronic lymphocitic leukemia; CYC, cyclophosphamide; DAA, direct antiviral agents; ELB, elbasvir; EPI, epidoxorubicin; ETO, etoposide; GLE, glecaprevir; GR, grazoprevir; HCV, hepatitis C virus; HSCT, hematopoietic stem cell transplantation; IMA, iImatinib; LDV, ledipasvir; LEU, leuprolide acetate; NIV, nivolumab; OXA, oxaliplatin; PAC, paclitaxel; PD, progressive disease; PEM, pembrolizumab; PER, pertuzumab; PIB, pibrentasvir; RBV, ribavirina; R-CHOP, rituximab+ cyclophosphamide + doxorubicin + vincristine + prednisone; SD, stable disease; SIM, simeprevir; SOF, sofosbuvir; SVR, sustained virological response; TAM, tamoxifen; TRA, trastuzumab; VEL, velpatasvir. * Fibrosis by fibroscan or clinical cirrhosis. ^ Oncologic outcome was evaluated 12 weeks after the end of DAA treatment.

## Data Availability

The data presented in this study are available on request from the corresponding author. The data are not publicly available due to patient privacy protection.
